# A Rare Tick Tale: A Novel Case of the Australian Paralysis Tick Causing Multiple Cranial Neuropathies

**DOI:** 10.1155/2024/3449614

**Published:** 2024-06-26

**Authors:** Sujan A. Surendran, Philomena McNamara, Jonathan N. Hyer, Charles S. Su

**Affiliations:** Department of Ophthalmology Royal Victorian Eye and Ear Hospital, Melbourne, Victoria, Australia

**Keywords:** Australian paralysis tick, cranial neuropathies, *Ixodes holocyclus*, orbital apex syndrome, tick bite

## Abstract

The Australian paralysis tick (*Ixodes holocyclus*) is found along the east coast of Australia. Tick bites may result in paralysis ranging from muscular weakness to ascending paralysis requiring respiratory support. Ocular complications and facial nerve involvement are rare. We present a rare occurrence of tick-bite-associated visual loss, proptosis, and multiple cranial neuropathies not previously reported in the literature. The tick was removed, and the patient's symptoms improved following treatment with steroids and oral doxycycline. The vision and sensory changes are not explained by the *Ixodes* toxin; thus, we hypothesize this is related to orbital apex inflammation.

## 1. Case Details

**Figure 1:**
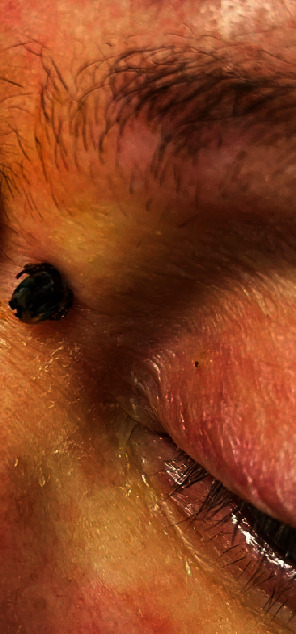
A dead *Ixodes holocyclus* tick was seen on the medial subbrow skin.

**Figure 2:**
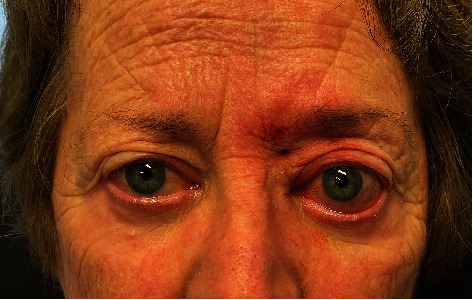
Unilateral proptosis, reduced orbicularis tone, and soft tissue inflammation (postremoval).

A 78-year-old female presented to the Royal Victorian Eye and Ear Hospital (RVEEH), Melbourne, Australia, with a 4-day history of left eye pain, proptosis, and reduction in vision after noting a lesion on the left medial canthus. She had a past ocular history of primary open-angle glaucoma. On examination, a dead *Ixodes holocyclus* tick was seen on the medial subbrow skin with minimal surrounding edema and no cellulitis (Figure 1). This tick had a typical appearance for *Ixodes holocyclus* species and was identified independently by a number of senior clinicians. Her visual acuity (VA) was reduced to 20/200 with a left relative afferent pupillary defect. There was 4 mm of left proptosis with decreased left periocular orbicularis oculi tone (Figure 2). She was unable to close the left eye (7 mm lagophthalmos). She had an abduction deficit (−0.5) and trigeminal nerve (V1, V3) paresthesia. Confrontational visual fields were intact in all four quadrants in both eyes. Anterior and posterior segment eye examinations were normal. The patient was systemically well, with no respiratory distress. A full neurological exam was conducted, which did not identify any other neurological deficits. Contrast-enhanced computed tomography (CT) of the orbits demonstrated preseptal soft tissue swelling with no significant inflammation of the orbital fat or extraocular muscles, and the orbital apex and cavernous sinus appeared normal. Due to the presence of a radio-opaque foreign object on the CT adjacent to the left lateral canthus, possibly representing a previously migrated glaucoma drainage device, an MRI could not be performed safely during her admission. The tick was removed, and she was treated with oral doxycycline and intravenous methylprednisolone with oral prednisolone taper. At 1-month follow-up with her local ophthalmologist, she had resolution of periocular sensation, ocular motility, and vision (VA 20/20). Proptosis and orbicularis weakness had improved but not been resolved.

## 2. Discussion

Paralysis in *Ixodes* bites is due to toxin release. The secreted neurotoxin, holocyclotoxin, is thought to interfere with presynaptic calcium influx at the neuromuscular junction, resulting in reduced acetylcholine release and muscle contraction [1]. The decreased tone of orbicularis oculi could be explained by the effect of this toxin. This mechanism does not, however, fully explain the findings of optic neuropathy and trigeminal sensory deficit in our patient. The widespread involvement of both motor and sensory nerves suggests localization to the orbital apex, similar to the Tolosa–Hunt syndrome [2]. *Ixodes holocyclus* has been reported to cause facial nerve paralysis and ocular motility deficits, but this is the first report of orbital apex syndrome secondary to a tick bite [3, 4].

There were no systemic or clinical concerns for tick-borne diseases such as rash, fevers, chills, headaches, arthropathies, and lymphadenopathy. Consequently, human tick-borne diseases known to be endemic to areas of the patient's travel, including Lyme disease, Q fever, spotted fever, tick typhus, and babesiosis, were not serologically tested [5]. Invasive CSF sampling and analysis were not performed to investigate other causes of cranial neuropathies, such as polyradiculitis cranialis, as the patient had shown signs of clinical improvement and reassuringly did not demonstrate other neurological deficits or respiratory compromise. Additionally, MRI and electromyography studies were not performed in the acute setting, but perhaps, if performed, they could have given insight into the extent of cranial nerves involved and the paralytic effects of the *Ixodes* toxin.

Management of tick bites includes prevention of bites, prompt removal, and monitoring. In paralysis tick-endemic areas, preventative methods include the use of insect repellents and regular body and clothing checks [6]. If bitten, it is important to remove the tick without scratching or pulling at live ticks to reduce toxin release. Antiserums and vaccines are used in animals, but their safety has not been studied in humans [7].

## 3. Conclusion

Our case demonstrates a previously unreported complication of a tick bite causing an inflammatory process, resulting in multiple cranial neuropathies localizing to the apex of the orbit. It highlights the importance of prompt removal of ticks and monitoring for complications following a tick bite. Steroid use may be useful to reduce inflammation and hasten resolution.

## Data Availability

The authors have nothing to report.
